# Ballistic Performance of 7A52/7A62 Aluminum Alloy Laminates: A Numerical Investigation of Configuration Effect

**DOI:** 10.3390/ma19010179

**Published:** 2026-01-03

**Authors:** Qunjiao Wang, Meilin Yin, Jiangong Zhou, Xinyu Liu, Hui Zhang, Ruibin Mei, Zejun Chen, Yu Cao, Qiang Wang, Fuguan Cong, Yunlong Zhang

**Affiliations:** 1 Key Laboratory of Electromagnetic Processing of Materials (Ministry of Education), Northeastern University, Shenyang 110819, China; yinmeilin0303@163.com (M.Y.); zhoujiangong_off@163.com (J.Z.); liuxinyu0016@163.com (X.L.); hzhang@epm.neu.edu.cn (H.Z.); 2 State Key Laboratory of Rolling and Automation, Northeastern University, Shenyang 110819, China; meiruibin@neuq.edu.cn; 3 International Joint Laboratory for Light Alloys, Ministry of Education, College of Materials Science and Engineering, Chongqing University, Chongqing 400044, China; zjchen@cqu.edu.cn (Z.C.); yucao928@cqu.edu.cn (Y.C.); 4 Northeast Light Alloy Co., Ltd., Harbin 150060, China; wangqiang@chinalco.com.cn (Q.W.); cfgdy2010@163.com (F.C.); zyl030202@126.com (Y.Z.)

**Keywords:** aluminum alloy laminated plates, configuration design, Johnson-Cook constitutive models, numerical simulation, ballistic performance

## Abstract

This study presents a systematic numerical investigation into the ballistic performance of 7A52/7A62 aluminum alloy laminated plates with varying configurations. The dynamic mechanical behavior of the base alloys, 7A52 and 7A62, was first characterized experimentally, and the corresponding Johnson-Cook (J-C) constitutive parameters were calibrated. Using the calibrated J-C model, a series of numerical simulations were performed on several structural configurations, including single-layer (7A52-A, 7A62-B), double-layer (AB, BA), and four-layer laminates (ABAB, BAAB, ABBA, BABA). The results demonstrate that four-layer laminates exhibit markedly better ballistic performance than monolithic and double-layer plates. Among them, the ABAB stacking sequence—arranged in an alternating soft–hard–soft–hard pattern—shows the optimal performance, yielding a residual projectile velocity of only 256 m/s. This represents an approximately 27% reduction compared to the monolithic high-strength 7A62 plate. The overall ranking of ballistic performance is as follows: ABAB > BAAB > ABBA > BABA. Energy-based analysis further indicates that multi-interface delamination, coupled with plastic deformation and damage evolution, improves the energy-absorption efficiency of the laminated plates and thus enhances their ballistic resistance. This study offers valuable guidance for the lightweight design of laminated 7XXX-series aluminum alloy protective plates.

## 1. Introduction

Aluminum alloys are widely employed in the military industry owing to their high specific strength, good machinability, and excellent electrical and thermal conductivity. These properties make them particularly suitable for applications in specialized vehicles, light and medium tanks, and aircraft [1,2,3,4,5,6]. In modern combat environments, personnel are primarily threatened by fragmentation from explosive blasts and impacts from small-caliber projectiles. Therefore, improving the protective performance of armored vehicles against blast, fragmentation, and penetration has become a critical objective to enhance personnel survival.

In the field of armor materials, aluminum alloys are mainly classified into the 2xxx, 5xxx, and 7xxx series. Among these, the 7xxx series offers substantially higher strength than other series, albeit with relatively lower fracture toughness [7,8,9,10]. Statistical analysis of ballistic limit velocities under impact by 7.62 mm APM2 projectiles indicates that the 7xxx series provides the best ballistic performance, followed by the 2xxx series, with the 5xxx and 6xxx series exhibiting progressively lower performance [11]. This trend highlights that material strength is a key factor governing the penetration resistance of aluminum alloys against rigid, sharp-nosed projectiles. Compared with conventional metallic armor, laminated panels made of 7xxx series aluminum alloys offer advantages such as low density, high ductility, excellent specific strength, considerable design flexibility, and superior energy absorption capacity. Their adaptability to complex battlefield environments can effectively enhance the survivability of armored vehicles.

Compared to monolithic aluminum alloy plates, multi-layered laminates fabricated via lamination processes exhibit not only refined grain structures but also multi-tier interfacial architectures. This distinctive structural characteristic enables the laminate to dissipate a greater amount of energy during fracture, thereby improving the penetration resistance of protective panels [12,13,14]. Such laminated configurations effectively combine the high strength of high-alloy aluminum alloys with the superior ductility of low-alloy aluminum alloys. Layered metal composites constitute a special category of composite materials, consisting of alternately stacked metal layers or metal layers with distinct interfaces [15]. These layered architectures play a critical role in enhancing fracture toughness [16,17], damage tolerance [18], impact resistance [19], damping capacity [20], and fatigue performance, while also improving the formability and ductility of inherently brittle materials [21]. Existing studies have demonstrated that aluminum alloy laminates achieve synergistic enhancement in mechanical properties beyond those of their constituent materials [16,22,23,24,25]. For instance, Alic and Danesh observed R-curve behavior in aluminum alloy laminates, which is not present in monolithic plates [26]. Research by B. Tekyeh et al. indicated that, compared to monolithic plates, aluminum alloy laminates exhibit significantly improved impact toughness, a property that can be further enhanced by increasing the number of layers and optimizing interfacial bonding strength [22]. Jimene et al. proposed that the toughening mechanisms in such laminates originate from interfacial debonding, crack re-nucleation in adjacent layers, and plastic deformation energy dissipation in ductile layers. This model suggests that interfacial debonding alleviates triaxial stress at crack tips and redistributes local stresses, while crack re-nucleation enhances toughness by consuming additional energy required for new crack initiation [20,23,24,26]. Collectively, these findings establish aluminum alloy laminated structures as promising candidates for lightweight armor systems. Therefore, a comprehensive understanding of the high-velocity impact response of aluminum alloy laminates with varying configurations is essential to advance their practical engineering application.

The Johnson-Cook (J-C) model has gained widespread acceptance in the field of metal penetration prediction owing to its intuitive calibration procedure, relatively few parameters, compatibility with most finite element software, and its capacity to simulate both local and global structural responses simultaneously [27,28,29,30,31,32]. In previous studies [28,30,31,32,33], researchers have employed the J-C model to characterize the flow and fracture behavior of projectiles impacting target plates, successfully conducting numerical simulations of penetration and perforation processes. For instance, Borvik et al. [27,34] modified the strain rate sensitivity expression [27] and incorporated a damage term into the J-C flow stress model to predict plugging failure in 12 mm thick Weldox 460 E steel plates impacted by a blunt projectile. Their results demonstrated that the model could accurately reproduce the ballistic limit velocity, with errors confined within 10% of experimental values, while effectively capturing the principal response and physical behavior of the target material. Similarly, Iqbal et al. [28] performed finite element simulations using LS-DYNA to evaluate the ballistic performance of 12 mm and 16 mm thick low-carbon steel plates against 7.62 mm armor-piercing projectiles at various impact angles, including ricochet conditions. Following material characterization and parameter calibration to obtain J-C flow stress and fracture model parameters, they validated the calibrated properties through numerical simulations of high-strain-rate stress tests. The results showed strong agreement between the numerical stress–strain curves and experimental data, and the model accurately predicted target failure modes, residual projectile velocities, and critical ricochet angles. In another study, Khare et al. [35] used Abaqus combined with digital image correlation to determine the J-C material and damage parameters for a novel armor steel, and subsequently conducted LS-DYNA simulations of 7.62 × 51 mm NATO standard ammunition impacting a 6 mm thick armor-grade steel plate. The simulations showed good agreement with experimental observations [30]. Collectively, these studies confirm that the Johnson-Cook flow stress and fracture models are effective in predicting material flow and fracture under impact and penetration conditions involving complex stress states, high strain rates, and temperature effects.

Although the purely empirical nature and uncoupled formulation of the Johnson–Cook (J-C) model have attracted some criticism for lacking physical coupling, its decoupled structure offers significant practical advantages by allowing flexible adjustment of modeling complexity. Moreover, conducting repeated ballistic limit (V_50_) tests for each configuration would entail preparing numerous laminates with different stacking sequences and performing multiple impact experiments, which poses considerable challenges in terms of time, cost, and material resources. Additional practical constraints associated with ballistic testing—such as limits on target size, high experimental costs, and difficulties in real-time projectile velocity measurement—further justify the use of numerical simulation at this stage. Therefore, we adopted the classical Johnson–Cook model to characterize the dynamic mechanical behavior of the materials [29] and first employed numerical simulation to investigate the penetration resistance of 7A52/7A62 aluminum alloy laminates with different configurations. This approach provides clear guidance for the subsequent design and validation of ballistic experiments.

Nevertheless, how the stacking sequence influences the ballistic impact behavior of layered 7xxx aluminum alloys remains inadequately understood. While prior studies have primarily compared the performance of single-layer, double-layer, or limited multi-layer systems [1,11,13,14], this work systematically examines 7A52/7A62 aluminum alloy laminates by varying the stacking sequence, thereby elucidating the effects of different configurations and the resulting multi-layer interfaces on damage evolution and energy-dissipation mechanisms under impact. The findings provide valuable insights for the design of ballistic-resistant aluminum alloy laminated plates.

## 2. Materials and Methods

### 2.1. Materials

This investigation employed three aluminum alloys: 7A52, 7A62, and 7A01. The chemical compositions of these alloys are presented in Table 1.

### 2.2. Configuration Design

**Figure 1:**
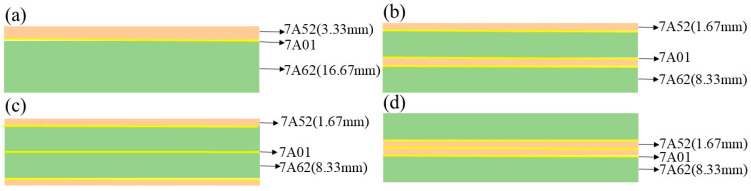
Configuration Design of Laminated Plates (**a**) AB/BA; (**b**) ABAB/BABA; (**c**) ABBA; (**d**) BAAB.

In this study, the laminated plates employ the more ductile 7A52 alloy and the higher-strength 7A62 alloy as the core substrates. To optimize interfacial bonding between these substrates and enhance the interlayer composite stability, a more ductile 7A01 aluminum alloy was introduced as an intermediate bonding layer. The excellent ductility and adhesion characteristics of 7A01 promote effective bonding between the core layers. The total thickness of the laminated plate is 20 mm, with a thickness ratio of 1:5 between the 7A52 (designated as layer A) and 7A62 (designated as layer B) core substrates. The specific configurations are shown in Figure 1.

### 2.3. Experimental Methods

The Johnson-Cook (J-C) model parameters for the 7A52 and 7A62 aluminum alloys were calibrated through a set of specifically designed mechanical tests. Quasi-static tensile tests at room temperature characterized the strain hardening; dynamic tensile tests and SHPB experiments quantified the strain rate sensitivity; and elevated-temperature tensile tests determined the thermal softening response.

**Figure 2:**
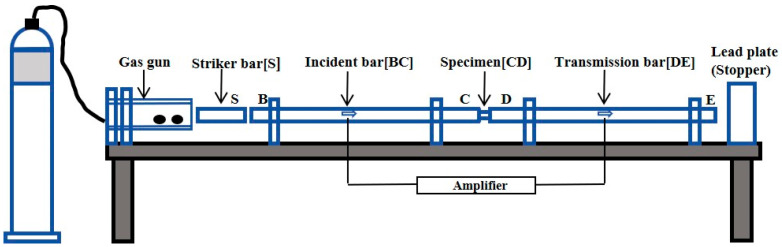
Schematic diagram of the Split Hopkinson Pressure Bar (SHPB) apparatus.

**Figure 3:**
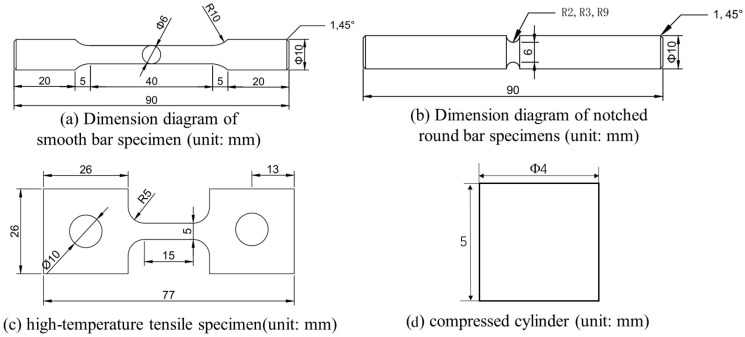
Geometric dimensions of specimens used in mechanical performance tests. (**a**) Dimension diagram of smooth bar specimen; (**b**) Dimension diagram of notched round bar specimens; (**c**) high-temperature tensile specimen; (**d**) compressed cylinder.

Tensile tests were conducted using an electronic universal testing machine at a strain rate of 1.5 mm/min, with strain data continuously acquired through an extensometer. Dynamic compression experiments were performed using a split Hopkinson pressure bar (SHPB) system (as shown in Figure 2) to investigate the dynamic mechanical response of the materials under high-strain-rate impact loading. The geometric dimensions of the specimens used, including smooth cylindrical, notched cylindrical, high-temperature tensile, and dynamic compression cylindrical specimens, are presented in Figure 3.

## 3. Material Model and Parameter Calibration

### 3.1. Johnson-Cook Constitutive Model

In 1983, Johnson and Cook [29], building upon the frameworks of continuum damage mechanics and viscoplasticity theory [36,37], systematically investigated the influences of high strain rate, temperature, and large plastic deformation on material behavior, and subsequently proposed the classic constitutive model known as the Johnson-Cook (J-C) model. The flow stress in the J-C model is defined as the product of three separate terms: the strain hardening term $\left(A\;+B{\varepsilon_{p}}^{n}\right)$, the strain rate hardening term $\left(1+C\ln\frac{\dot{\varepsilon }}{{\dot{\varepsilon }}_{0}}\right)$, and the thermal softening term $\left(1-\left[\frac{T-T_{room}}{T_{melt}-T_{room}}\right]\right)$. The full constitutive equation is expressed as:

(1) $$\sigma =\left(A+B{\varepsilon_{p}}^{n}\right)\left(1+C\ln\frac{\dot{\varepsilon }}{{\dot{\varepsilon }}_{0}}\right)\left(1-{\left[\frac{T-T_{room}}{T_{melt}-T_{room}}\right]}^{m}\right)$$

where *A*, *B*, *n*, *C* and *m* are the constitutive model parameters; *A* represents the quasi-static yield stress at room temperature; *B* is the strain hardening coefficient; $\varepsilon_{p}$ denotes the equivalent plastic strain; *n* is the strain hardening exponent; *C* characterizes the strain rate sensitivity; *m* is the thermal softening exponent; σ is the equivalent flow stress; $\dot{\varepsilon }$ is the current strain rate; ${\dot{\varepsilon }}_{0}\;=$ 1 × 10^−3^s^−1^ is the reference strain rate; *T* is the current temperature; $T_{room}$ is the reference room temperature; and $T_{melt}$ is the material’s melting temperature. The dimensionless strain rate is defined as $\frac{\dot{\varepsilon }}{{\dot{\varepsilon }}_{0}}\;=\varepsilon^{*}$, and the dimensionless temperature as $\frac{T-T_{room}}{T_{melt}-T_{room}}\;={\;T}^{*}$.

#### 3.1.1. Constitutive Model Strain Parameter Calibration

For smooth round bar specimens under quasi-static tensile testing at room temperature, both the strain rate hardening term and the thermal softening term in Equation (1) become unity. Consequently, the constitutive model reduces to the simplified form given by Equation (2):

(2) $$\sigma =A+B\varepsilon^{n}$$

**Figure 4:**
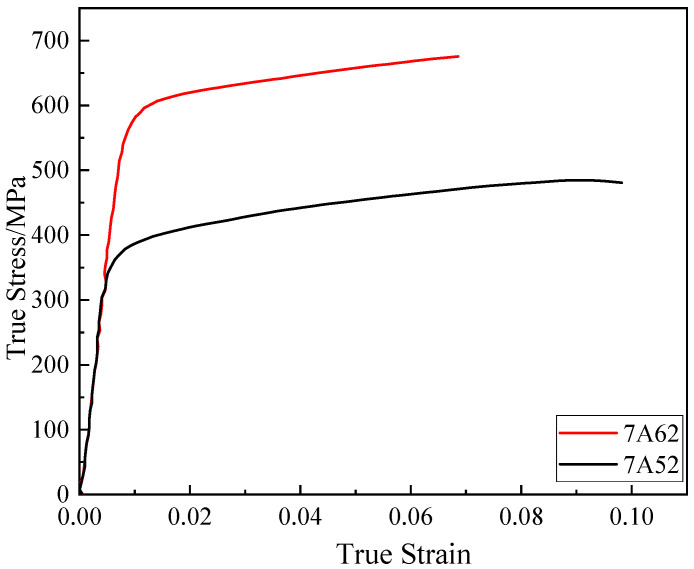
Quasi-static tensile true stress–strain curve.

Figure 4 displays the true stress–strain curves acquired from quasi-static tensile tests performed on smooth round bars at room temperature. The yield strength *A* is taken as the true stress at 0.2% plastic strain. The strain-hardening coefficient *B* and the hardening exponent *n* were obtained by fitting the true stress–strain data between the yield point and the onset of necking. To ensure continuity of the constitutive model (Equation (2)) at the yield point, the fitting of *B* and *n* was constrained such that the stress value of the fitted curve at zero equivalent plastic strain ($\varepsilon_{p}$ = 0) equals the yield strength *A*. The curve fitted using experimental data near the yield point ($\varepsilon_{p}$ = 0.002) extrapolates to a stress value at zero strain that matches *A*, thereby confirming the continuity of the parameter set (*A*, *B*, *n*). The determined stress triaxiality and fracture strain values for both alloys are listed in Table 2. The calibrated parameters are summarized in Table 3.

#### 3.1.2. Constitutive Model Strain Rate Parameter Calibration

**Figure 5:**
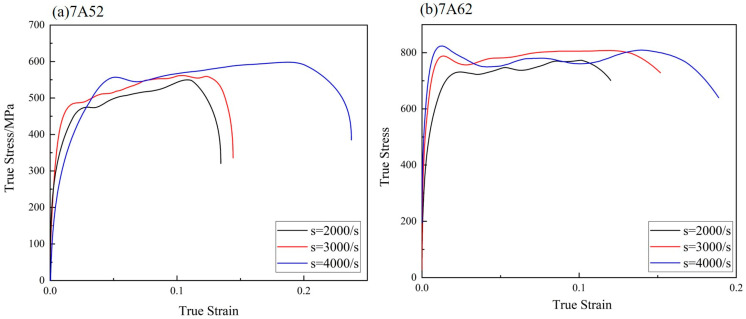
Stress–Strain Curves of Two Alloys under High-Speed Impact.

To evaluate the strain rate sensitivity of the 7A52 and 7A62 alloys, true stress–strain curves were acquired at various strain rates using a Hopkinson bar apparatus, as presented in Figure 5. The results indicate that both the yield strength and the fracture strain of the two alloys increase with the strain rate.

Under dynamic loading at room temperature, the initial yield condition corresponds to $\varepsilon_{p\;}\;$= 0. Thus, the strain hardening term becomes *A*, and the thermal softening term is unity. Accordingly, the equivalent flow stress simplifies to the form in Equation (3):

(3) $$\sigma =A\left(1+C\ln\frac{\dot{\varepsilon }}{{\dot{\varepsilon }}_{0}}\right)$$

**Figure 6:**
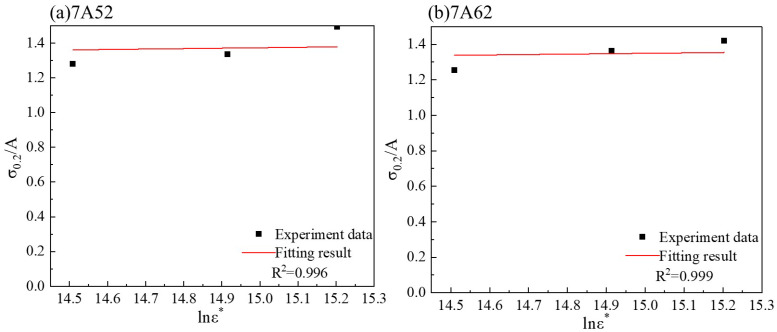
Fitting of Parameter *C*.

The strain rate sensitivity coefficient *C* was determined by fitting Equation (3) to the measured yield strengths at different strain rates, as shown in Figure 6. The resulting value of *C* is listed in Table 3.

#### 3.1.3. Calibration of the Temperature Term Parameters in the Constitutive Model

**Figure 7:**
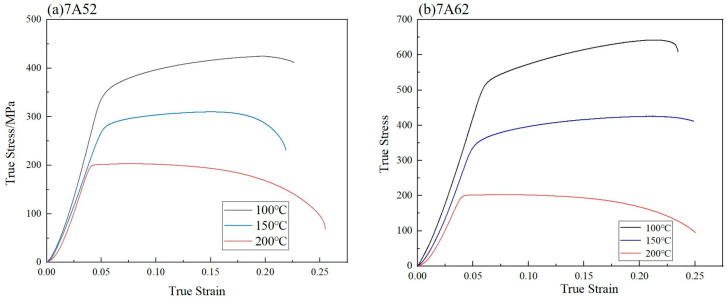
True Stress–Strain Curves of Two Aluminum Alloys under High-Temperature Tensile Tests.

**Figure 8:**
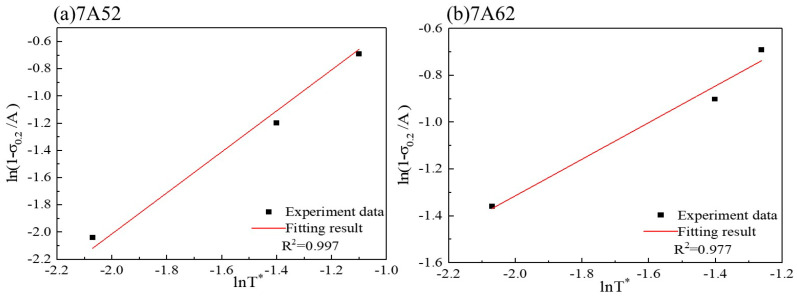
Fitting of Parameter *m* for the Two Aluminum Alloys.

Based on the quasi-static high-temperature tensile test conditions, the strain rate term in the Johnson–Cook (J-C) model is taken as unity. The resulting expression for equivalent flow stress under these conditions is given by Equation (4). At the initial yield point under quasi-static loading, where the plastic strain is zero, Equation (4) can be rearranged to yield Equation (5). As illustrated in Figure 7 the yield strength decreases markedly with increasing temperature, reflecting a significant thermal softening effect in both the 7A52 and 7A62 aluminum alloys. Finally, by applying Equation (5) to fit the experimental data shown in Figure 8 the thermal softening coefficient *m* is determined, and the resulting values are summarized in Table 3.

(4) $$\sigma =\left(A+B{\varepsilon_{p}}^{n}\right)\left(1-{\left[\frac{T-T_{room}}{T_{melt}-T_{room}}\right]}^{m}\right)$$

(5) $$\ln\left(1-\frac{\sigma_{0.2}}{A}\right)=mln\frac{T-T_{room}}{T_{melt}-T_{room}}$$

### 3.2. J-C Fracture Criterion

The fracture criterion initially developed by Hancock and Mackenzie was subsequently extended by Johnson and Cook [38] to incorporate the effects of stress triaxiality, temperature, strain rate, and strain path on the fracture strain. In this model, damage is assumed to accumulate progressively in the material during plastic deformation, with failure occurring abruptly once the damage reaches a critical threshold. The failure criterion is expressed as:

(6) $$\varepsilon_{f}=\left[D_{1}+D_{2}e^{D_{3}\eta }\right]\left[1+D_{4}\ln\left(\frac{\dot{\varepsilon }}{{\dot{\varepsilon }}_{0}}\right)\right]\left[1+D_{5}\left(\frac{T-T_{room}}{T_{melt}-T_{room}}\right)\right]$$

where $\varepsilon_{f}$ is the equivalent fracture strain under the current stress state, temperature, and strain rate; *D*_1_, *D*_2_, and *D*_3_ are parameters characterizing the dependence of fracture strain on stress triaxiality; *D*_4_ is the strain rate sensitivity coefficient for fracture; and *D*_5_ is the thermal sensitivity coefficient governing the temperature dependence of fracture strain.

#### 3.2.1. Calibration of Stress State Parameters in the Fracture Criterion

To investigate the influence of stress triaxiality on the fracture criterion, quasi-static tensile tests were performed at room temperature using notched round bar specimens with varying notch radii (where a smooth specimen corresponds to an infinite notch radius). Under these quasi-static and room-temperature conditions, the strain rate and temperature terms in the Johnson–Cook model reduce to unity. Accordingly, the fracture criterion simplifies to the following expression:

(7) $$\varepsilon_{f}=D_{1}+D_{2}e^{D_{3}\eta }$$

In this expression, *η* denotes the stress triaxiality. For notched round bar specimens, Wang [39] proposed the following formula to calculate the initial stress triaxiality:

(8) $$\eta =\frac{1}{3}+\ln\left(1+\frac{a_{0}}{2R_{0}}\right)$$

where $a_{0}$ is defined as the initial minimum cross-sectional radius at the notch, and *R*_0_ is the initial notch root radius.

A defined relationship exists between stress triaxiality and fracture strain [40]. Stress triaxiality characterizes the triaxial stress state, while fracture strain corresponds to the critical strain at material failure. The fracture strain is determined by the following expression:

(9) $$\varepsilon_{f}=\ln\frac{A_{0}}{A_{f}}$$

with $A_{0}$ representing the initial minimum cross-sectional area of the specimen and $A_{f}$ the cross-sectional area after fracture. 

**Figure 9:**
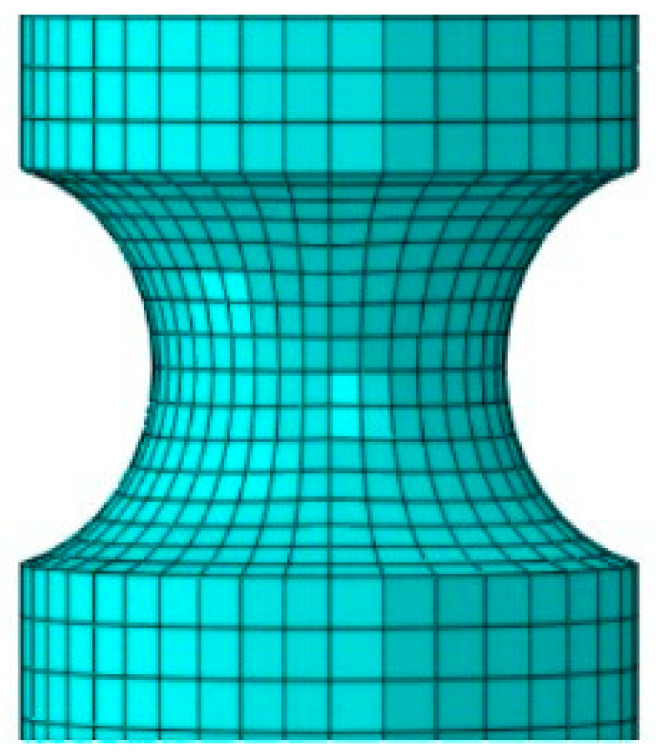
Local mesh refinement of the notched tensile specimen model.

**Figure 10:**
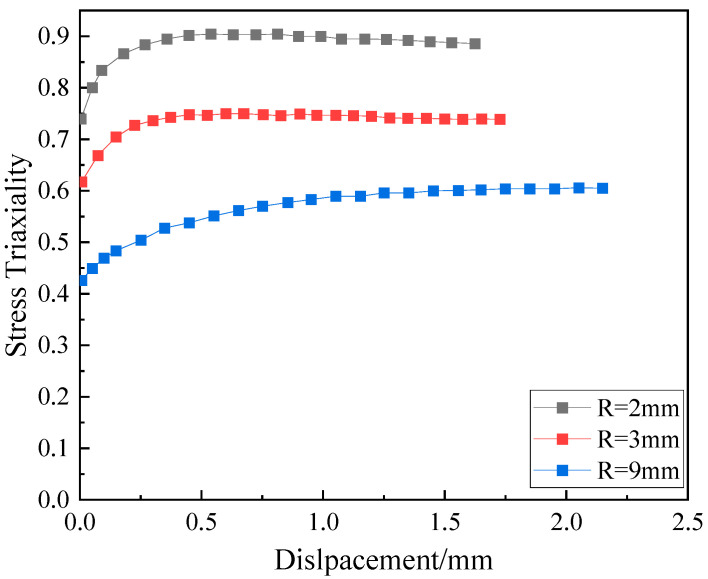
Curve of stress triaxiality versus displacement during the notched tensile process of 7A52 alloy.

Owing to the high ductility of the 7A52 alloy, significant deformation during loading leads to notable changes in the notch geometry after fracture. As a result, the stress triaxiality at fracture cannot be accurately determined using analytical formulas based on the initial notch radius. To address this, a tensile simulation of the notched cylindrical specimen was conducted using the explicit dynamics module in Abaqus. The model was discretized with C3D8R elements, with local mesh refinement applied at the notch region, achieving a minimum element size of 0.5 mm, as illustrated in Figure 9. The strain-dependent parameters of the Johnson-Cook constitutive model for the 7A52 aluminum alloy were incorporated into the simulation. The resulting load–displacement curve from the numerical analysis is presented in Figure 10.

**Figure 11:**
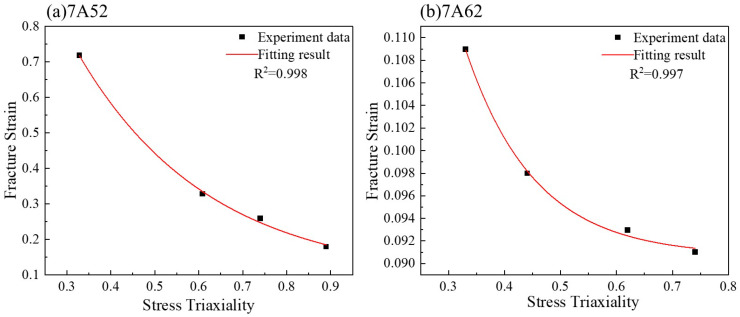
Obtaining *D*_1_–*D*_3_ parameters for the two types of alloys.

The relationship between the fracture strain of the notched specimen and the stress triaxiality was fitted using Equation (7), as shown in Figure 11. The obtained parameters *D*_1_, *D*_2_, and *D*_3_ are listed in Table 3.

#### 3.2.2. Calibration of the Strain Rate Parameter in the Fracture Criterion

**Figure 12:**
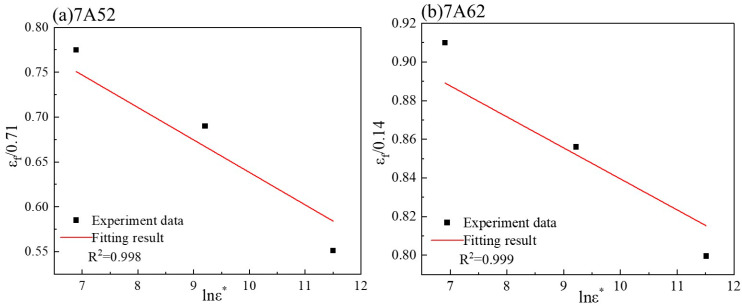
Obtaining *D*_4_ parameters for the two types of alloys.

**Figure 13:**
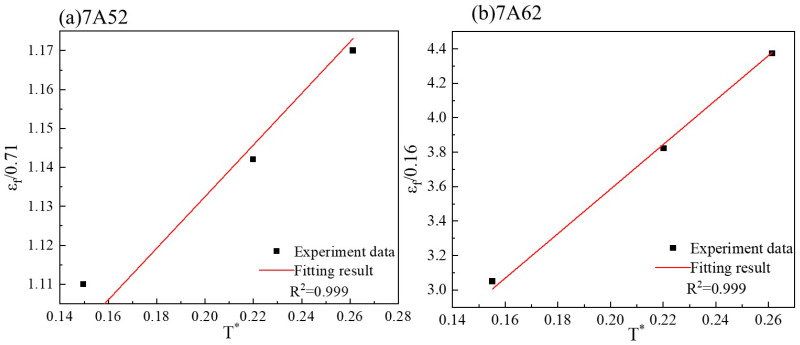
Obtaining *D_5_* parameters for the two types of alloys.

The fracture strain of specimens under dynamic loading at room temperature is determined using Equation (10). The fracture strain of tensile specimens is calculated via Equation (9), with a reference strain rate set to ${\dot{\varepsilon }}_{0}\;$= 1 × 10^−3^. The relationship between fracture strain and the dimensionless strain rate is illustrated in Figure 12. A linear fitting procedure based on Equations (11) and (12) was applied to the data in Figure 13, yielding the strain rate sensitivity parameter $D_{4}$, which is summarized in Table 3.

(10) $$\varepsilon_{f}=\left[D_{1}+D_{2}e^{D_{3}\eta }\right]\left[1+D_{4}\ln\left(\frac{\dot{\varepsilon }}{{\dot{\varepsilon }}_{0}}\right)\right]$$

when the stress triaxiality *η* = 0.33, for 7A52 alloy, the equation transforms to:

(11) $$\frac{\varepsilon_{f}}{0.71}=1+D_{4}\ln\varepsilon^{*}$$

For 7A62 alloy, the equation transforms to:

(12) $$\frac{\varepsilon_{f}}{0.14}=1+D_{4}\ln\varepsilon^{*}$$

#### 3.2.3. Calibration of Temperature Parameter in the Fracture Criterion

Figure 7 presents the true stress–strain curves obtained from quasi-static tensile tests at elevated temperatures. As shown, the fracture strain of both 7A52 and 7A62 alloys increases progressively with temperature. Under quasi-static conditions, the strain rate term is taken as unity, and the fracture strain is described by Equation (13). The fracture strain values of the high-temperature tensile specimens were calculated using Equation (9), and their variation with dimensionless temperature is plotted in Figure 13. A linear fitting procedure based on Equations (14) and (15) was applied to the data in Figure 13, yielding the thermal sensitivity coefficient $D_{5}$, which is summarized in Table 3.

(13) $$\varepsilon_{f}=\left[D_{1}+D_{2}e^{D_{3}\eta }\right]\left[1+D_{5}\left(\frac{T-T_{room}}{T_{melt}-T_{room}}\right)\right]$$

Under a stress triaxiality of *η* = 0.33, the failure criterion for the 7A52 alloy becomes:

(14) $$\frac{\varepsilon_{f}}{0.71}=1+D_{5}T^{*}$$

Similarly, for the 7A62 alloy, the equation is given by:

(15) $$\frac{\varepsilon_{f}}{0.14}=1+D_{5}T^{*}$$

### 3.3. Validation of Parameter Effectiveness

**Figure 14:**
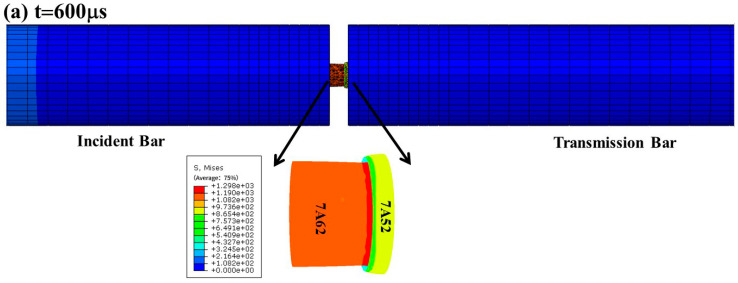

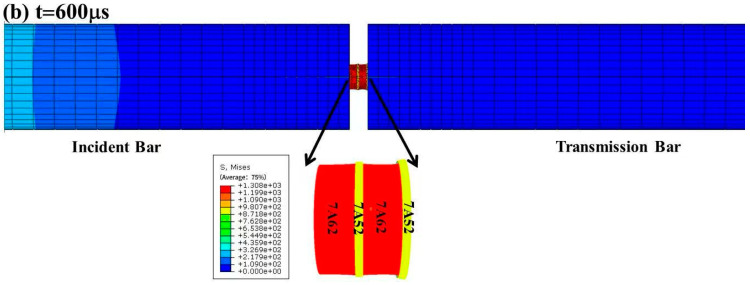
Stress distribution and macroscopic morphology of SHPB simulations for laminated plates with different configurations (Unit: MPa) (**a**) AB; (**b**) ABAB.

**Figure 15:**
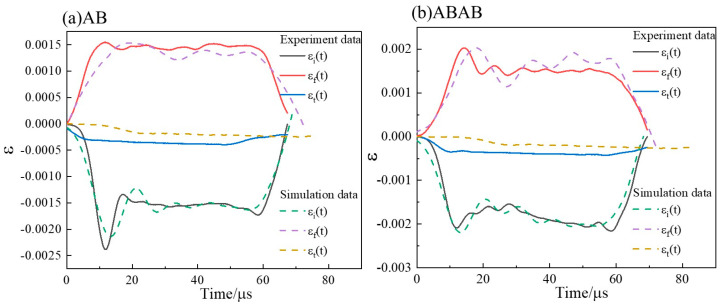
Comparison of stress–time curves from SHPB experiment and numerical simulation.

Validation of the calibrated Johnson–Cook parameters was carried out via split Hopkinson pressure bar (SHPB) experiments and matching numerical simulations on AB and ABAB laminates that include a 7A01 adhesive interlayer. For the simulation, the 7A01 layer was modeled as linear elastic, and the interfaces were represented using a surface-based bilinear cohesive zone model (CZM). The dynamic stress distribution from the SHPB simulation and a comparison of experimental and simulated strain–time curves are provided in Figure 14 and Figure 15, respectively. The good agreement between the two sets of curves confirms the validity of the parameters under high-strain-rate conditions and provides a reliable basis for the following ballistic simulations.

## 4. Results

### 4.1. Simulation Modeling

**Figure 16:**
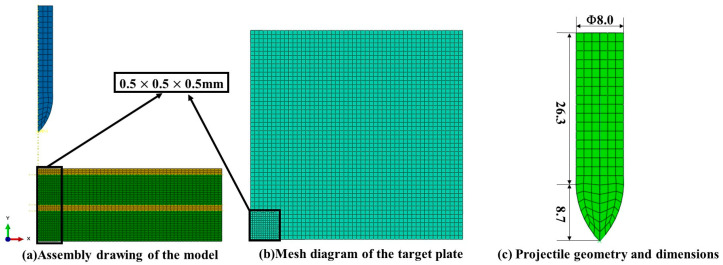
Finite Element Model.

To improve computational efficiency, a quarter-symmetric three-dimensional finite element model was established in ABAQUS/Explicit to simulate the projectile impact process. The plastic deformation and fracture behavior of the materials were described using the Johnson–Cook (J-C) constitutive model with the previously calibrated parameters. As shown in Figure 16, the target plate was modeled as an elastoplastic deformable body, while the projectile was treated as a rigid body with a mass density of 7.74 g/cm^3^. The projectile was discretized with a uniform mesh of element size 1 mm × 1 mm × 1 mm. Given the anticipated severe plastic deformation and fracture in the impact zone, the mesh in this region was locally refined. A mesh sensitivity study indicated that refining the element size in the impact zone to 0.5 mm essentially converged the predicted residual projectile velocity. Compared with a finer mesh of 0.25 mm, the 0.5 mm mesh provides sufficient computational accuracy while substantially reducing computational cost. Consequently, an element size of 0.5 mm was adopted for the impact region in this work. Both the target and the projectile were meshed with C3D8RT elements (8-node linear thermally coupled brick elements with reduced integration and hourglass control).

The Abaqus model utilized the general contact algorithm to define the interactions, encompassing both the projectile-target contact and the self-contact of the target plate material. The contact domain included the external surfaces as well as the internal surfaces adjacent to the impact region. Normal contact behavior was simulated via a “hard contact” pressure-overclosure relationship. For the tangential direction, a constant Coulomb friction coefficient of 0.1 was applied to the projectile-target interaction, in accordance with Ref. [1]. Displacement boundary conditions were applied to the nodes at the peripheral regions of the target plate to represent realistic constraints. The interface between the two material layers was simulated using a cohesive surface-based interaction approach, with its constitutive response governed by a bilinear traction-separation law.

The intermediate 7A01 aluminum alloy layer primarily functions as an adhesive to bond the 7A52 and 7A62 layers. Due to its relatively small thickness and limited role in carrying primary plastic deformation under high-velocity impact, its inherent mechanical behavior was simplified as linear elastic, with an elastic modulus of 70 GPa and a Poisson’s ratio of 0.33. Interface bonding and failure were characterized by a surface-based cohesive zone model employing a bilinear traction-separation law. The key parameters are as follows: both the normal and tangential interface strengths are 94 MPa, with a corresponding critical displacement of 0.1 mm [11]. This gives a calculated critical fracture energy of $G_{Ic}$ = $G_{IIc}\;$= 4.7 N/mm. The initial interface stiffness was set to $K_{nn\;}$=${\;K}_{ss}\;$= 1 × 10^5^ N/mm^3^. All parameters are summarized in Table 4.

### 4.2. Simulation Results

#### 4.2.1. Ballistic Performance

**Figure 17:**
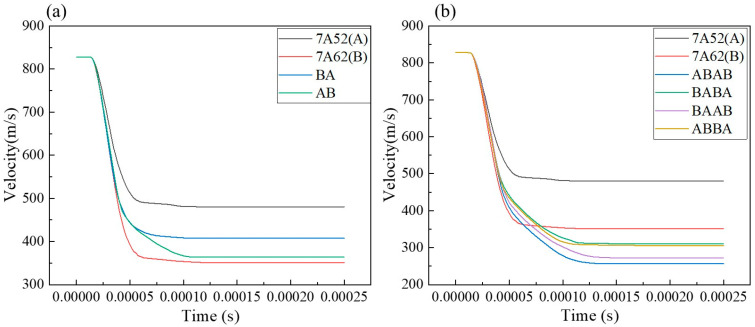
Variation in projectile velocity for laminates with different configurations: (**a**) Comparison between single-layer and double-layer plates; (**b**) Comparison between single-layer and four-layer plates.

The ballistic performance of 7A52/7A62 aluminum alloy laminates with different configurations was evaluated by analyzing the projectile’s velocity history during penetration. Under the same initial impact velocity, a faster deceleration of the projectile indicates better impact resistance of the target. The residual velocities for all configurations are listed in Table 5, and the corresponding velocity-time curves are plotted in Figure 17. The simulation results reveal that the ballistic performance of the two-layer configurations (AB and BA) lies between that of the single-layer plates A and B (Figure 17a). When the number of layers increases to four, the laminates outperform both the single-layer and double-layer configurations. As shown in Figure 17b, the impact resistance of the four-layer configurations ranks as follows: ABAB > BAAB > ABBA > BABA.

To provide preliminary validation, the predicted residual velocities in this study were compared with published gas-gun test data for 7A52/7A62-based 7XXX plates and laminates. Min et al. [11] reported ballistic experiments on 7A52-T6, 7A62-T6, and a 7B52 laminate (total thickness ≈ 20–21 mm) impacted by 6.0 mm projectiles with different nose shapes. Although the projectile geometry and impact conditions are not identical to those used here, the computed residual velocities for the monolithic plates (7A52: 480 m/s; 7A62: 351 m/s at 828 m/s) are of the same order of magnitude as the reported measurements, supporting the plausibility of the calibrated constitutive/failure parameters and the penetration modeling. What differs is that our results demonstrate that the ballistic benefit of lamination is configuration-dependent: a single-interface laminate does not necessarily outperform the best monolithic alloy at the same areal density, whereas multi-interface designs can provide greater velocity reduction, which is attributed to additional wave reflections, progressive delamination, and crack deflection. This trend is consistent with prior experimental and analytical observations on layered protective plates [12,13,14].

Although the predicted residual velocities for the monolithic plates show good agreement with available experimental data, the relative performance ranking among the multi-layer configurations has yet to be validated experimentally. Consequently, the present work mainly offers a numerical guideline to inform optimal design decisions at this stage.

#### 4.2.2. Deformation Evolution and Fracture Mechanism

**Figure 18:**
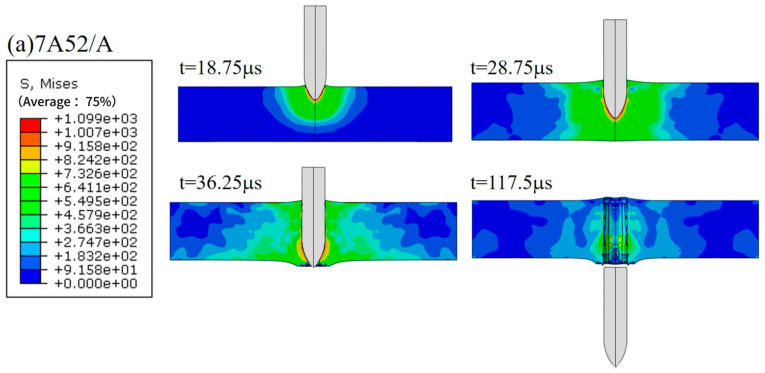

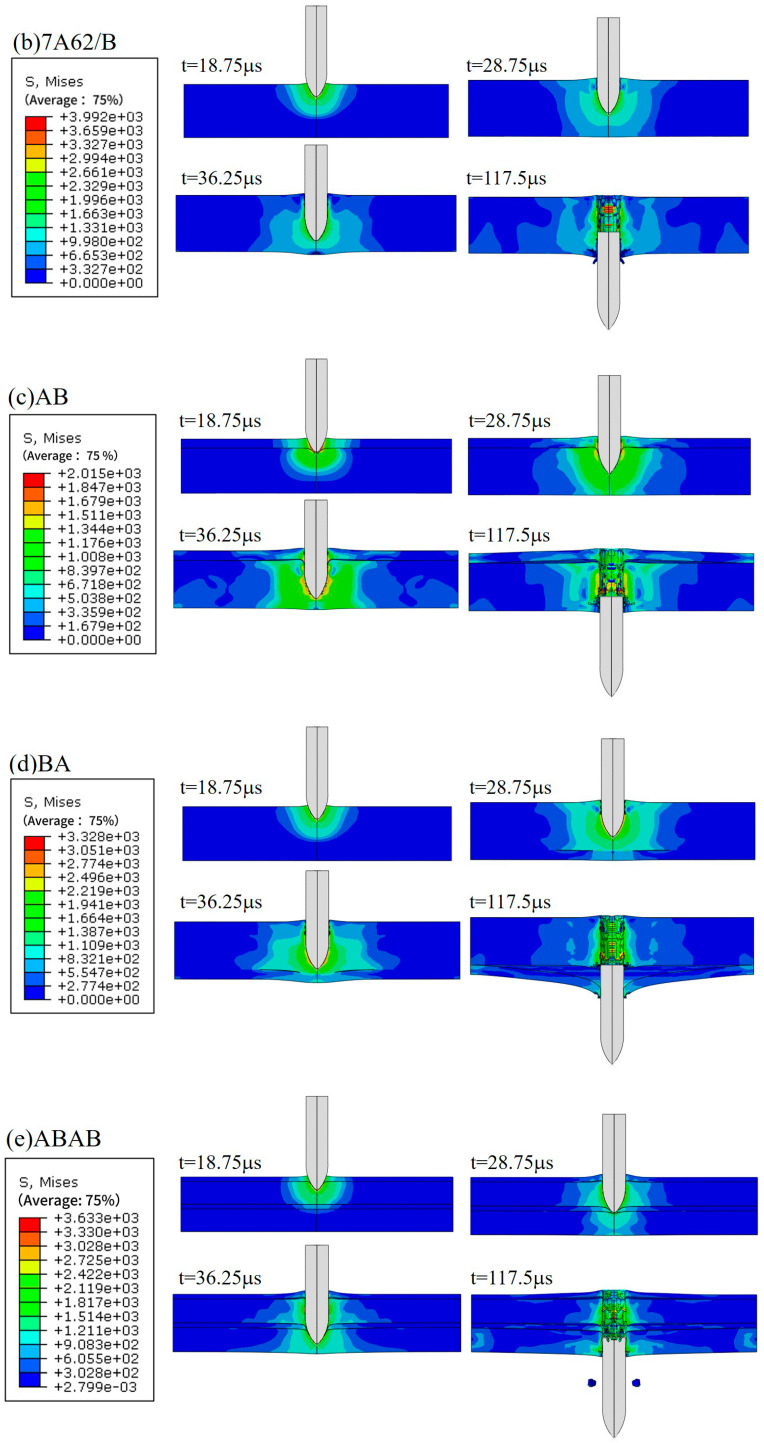

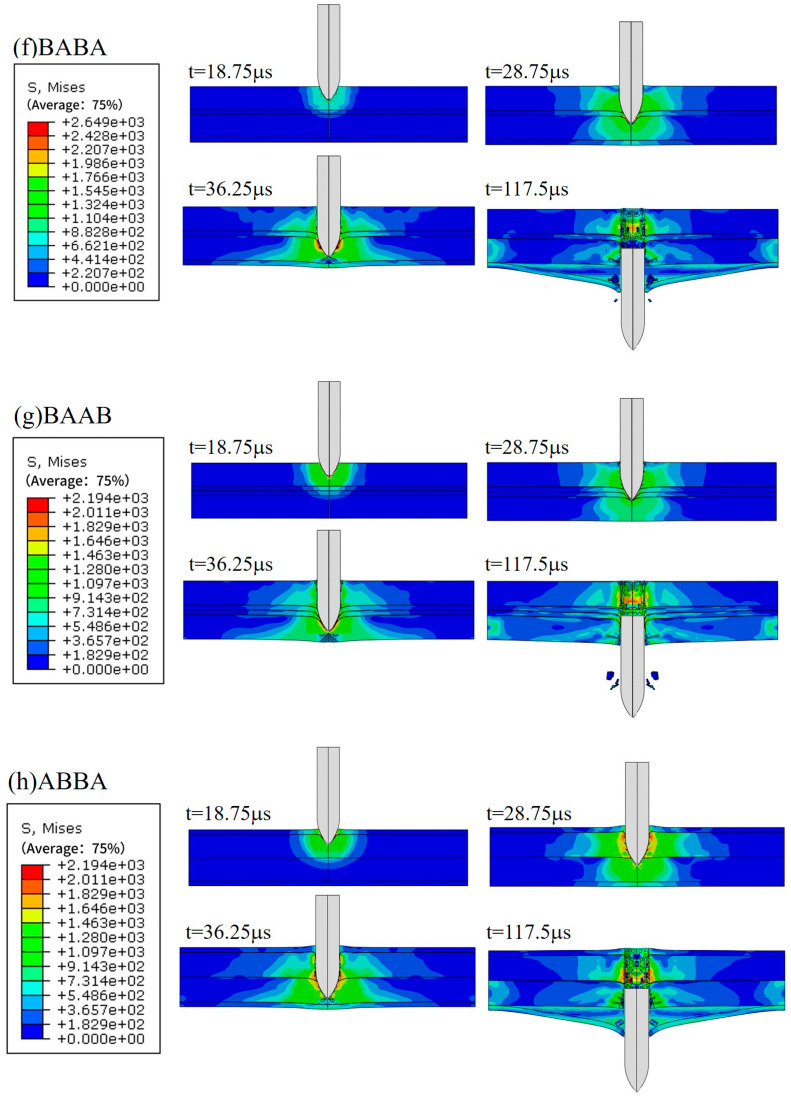
Mises Stress Distribution Contour Maps of Laminates with Different Configurations (Unit: MPa).

To elucidate the underlying mechanisms responsible for the differences in ballistic performance among various configurations, this section provides a comprehensive analysis combining stress evolution and failure modes. The simulations reveal a non-monotonic configuration effect: the performance of double-layer plates (AB/BA) lies between that of the monolithic plates A and B, whereas all four-layer configurations exhibit significantly superior performance, with an internal ranking of ABAB > BAAB > ABBA > BABA. The fundamental reason lies in the essential differences between laminated structures and monolithic materials in terms of stress-wave propagation and failure modes. As shown in Figure 18, stress waves propagate rapidly both through the thickness and laterally in monolithic plates. In laminated structures, however, the interlayer interfaces obstruct the propagation of stress waves (Figure 18c–g), weakening the through-thickness transmission of stress.

**Figure 19:**
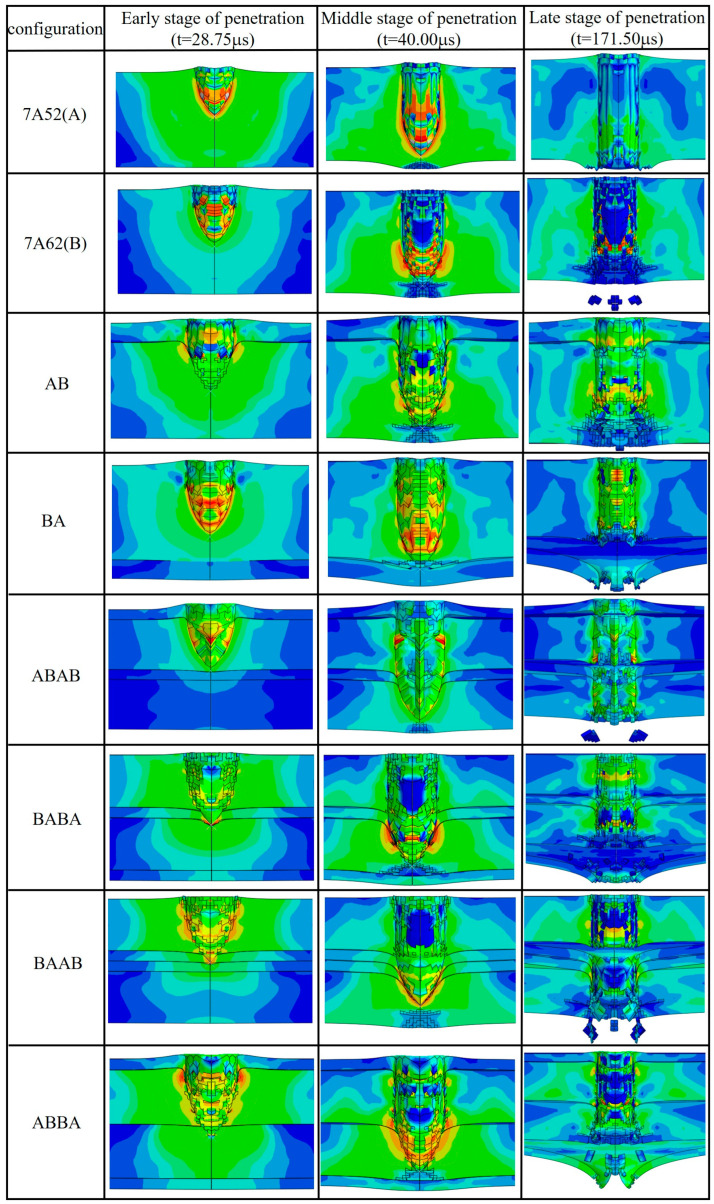
Failure Modes Throughout the Entire Penetration Process.

Under this framework, the inferior ballistic performance of the double-layer plates relative to monolithic plate B stems from their single interface being prone to premature debonding under high-velocity impact (Figure 19). Once the interface completely fails, the stress-wave transmission path is severed, and the front and rear layers lose their ability to act synergistically. The configuration effectively behaves as a monolithic plate with pre-damage (i.e., the debonded interface), whose overall stiffness and integrity are consequently lower than that of the intact monolithic plate B. In contrast, the four-layer plates achieve synergistic energy absorption through a “multi-interface progressive failure” mechanism, in which stress waves are repeatedly attenuated and damage is effectively dispersed. Different stacking sequences influence the final performance by governing the initial loading conditions of the stress waves and the initiation sequence of interface failures.

The optimal ABAB (soft–hard–soft–hard) configuration most significantly impedes stress-wave transmission during impact. Specifically, the first soft layer absorbs part of the kinetic energy through plastic deformation in the early stage of impact and alters the stress-wave morphology transmitted to the subsequent hard layer. The following hard layer then provides the primary penetration resistance. Subsequently, as the stress wave passes again through a soft layer and interface, further energy is dissipated via plastic deformation and interfacial debonding, while part of the stress wave is reflected, significantly mitigating stress concentration in the hard layer and thereby delaying its shear failure. The repetition of this process enables multi-stage energy dissipation. Because the acoustic impedances of 7A52 and 7A62 are relatively close, stress-wave reflection at each interface is moderate; therefore, the observed advantage of multi-interface configurations are attributed primarily to progressive delamination, crack deflection, and enhanced plastic deformation promoted by the additional interfaces. The performance of the BAAB (hard–soft–soft–hard) configuration lies between that of ABAB and ABBA. Its first layer is hard, providing high initial penetration resistance, followed by two consecutive soft layers that can absorb substantial energy through plastic deformation and attenuate the stress wave to some extent. However, the continuous distribution of soft layers somewhat diminishes the alternating soft–hard effect on stress-wave modulation. Moreover, the transition back to a hard layer at the rear tends to induce a back-face shear-failure mode. Thus, while its overall synergistic effect surpasses that of ABBA and BABA, it falls short of the alternating soft–hard structure of ABAB. In contrast, the weakest-performing BABA (hard–soft–hard–soft) configuration subjects the structure to more intense initial stress-wave loading due to its hard first layer. As a result, the subsequent soft layer fails to significantly modify the stress-wave morphology before it rapidly loads the second hard layer, leading to earlier stress concentration in the subsequent hard layers and under-utilization of the following materials and interfaces. Meanwhile, the ABBA (soft–hard–hard–soft) configuration benefits from the initial buffering effect of a soft impact face, which to some extent optimizes the stress-wave propagation path, resulting in slightly better synergy than BABA.

### 4.3. Energy Dissipation

Quantitative energy analysis provides further corroboration for the mechanisms outlined above. This study focuses on the energy absorption due to irreversible deformation of the target plate, categorized as plastic dissipation energy (from material plastic flow) and damage dissipation energy (from material damage and failure). The global energy balance was monitored throughout all simulations using the history outputs in Abaqus/Explicit. The initial kinetic energy of the projectile is primarily converted into: (i) its residual kinetic energy after perforation, (ii) the kinetic energy imparted to the target, and (iii) the internal energy of the deforming plates—supplemented by contributions from damage/cohesive dissipation and contact work. Artificial energies, such as hourglass and bulk-viscosity energy, were verified to remain at acceptably low levels relative to the total energy, confirming good numerical stability. The plastic dissipation and damage-related energies reported in Table 6 were extracted from the corresponding Abaqus history outputs once the energy curves stabilized at a plateau after complete perforation.

**Figure 20:**
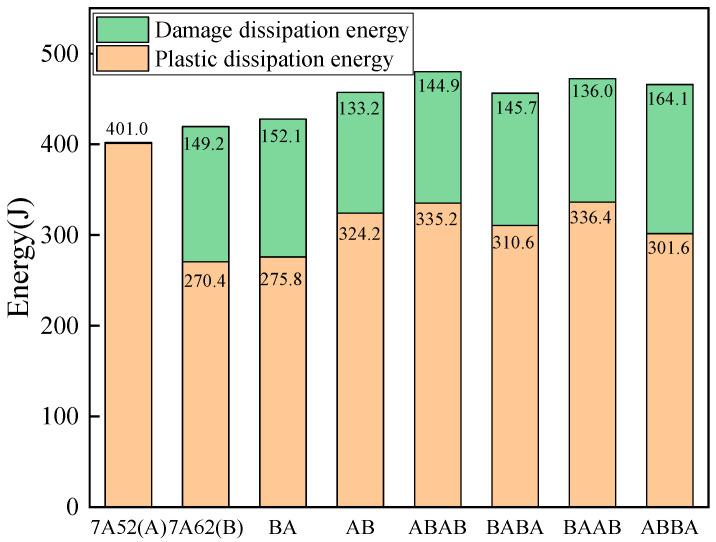
Energy Dissipation of Different Configurations.

As shown in Table 6 and Figure 20, the total energy dissipation—comprising both plastic and damage dissipation—for the four-layer configurations ranges from 456.3 J to 480.1 J. These values exceed those of the monolithic plates (A: 401.9 J; B: 419.2 J). The ABAB configuration shows the highest total energy dissipation (480.1 J), representing an increase of approximately 14.5% compared to the monolithic high-strength plate B (419.2 J).

Further analysis of the energy dissipation patterns shows that the more ductile monolithic plate A (7A52) is dominated by plastic dissipation, whereas the high-strength monolithic plate B (7A62) and the double-layer plates exhibit a mixed mode of plastic and damage dissipation. Notably, while maintaining relatively high damage dissipation, the four-layer configurations all show higher absolute plastic dissipation energy than monolithic plate B. This demonstrates that the four-layer plates, through optimized stacking sequences, successfully combine the ductility of the soft 7A52 layer with the high strength of the hard 7A62 layer, achieving the distinctive synergistic strengthening effect characteristic of multi-layer aluminum alloy laminates.

The divergence in energy dissipation pathways stems from the role of interlayer interfaces during impact. In alternately stacked configurations, these interfaces alter the propagation path of stress waves and blunt crack tips, thereby promoting more widespread and uniform plastic deformation in the ductile layers and enhancing plastic energy dissipation. At the same time, the interfaces can trigger crack deflection, branching, and secondary nucleation in neighboring layers, shifting the material’s damage evolution from a localized process to a progressive, multi-path dissipation mechanism. This mode of damage propagation effectively scatters the damage zone and increases the efficiency of damage-related energy absorption. By integrating multiple interfaces, the four-layer plate configurations achieve effective synergy among internal energy dissipation routes. The optimal ABAB configuration maximizes synergy between plastic and damage dissipation through its alternating “soft–hard–soft–hard” stacking sequence. In contrast, other configurations show a somewhat diminished capacity to harmonize these two mechanisms due to differences in layering design, leading to variations in overall performance.

## 5. Conclusions and Prospects

### 5.1. Conclusions

This study systematically investigates the configuration effects of 7A52/7A62 aluminum alloy laminated plates through numerical simulation, leading to the following main conclusions:

1. The mechanical behavior of 7A52 and 7A62 aluminum alloys was thoroughly characterized under varying stress states, strain rates, and temperatures. These results provided a reliable basis for calibrating the parameters of the Johnson-Cook flow stress and fracture models.
2. The four-layer configurations overall exhibit superior ballistic performance compared to monolithic and double-layer plates, with their performance ranked as follows: ABAB (256 m/s) > BAAB (272 m/s) > ABBA (305 m/s) > BABA (310 m/s). Among these, the optimal ABAB configuration achieves approximately a 27% reduction in residual velocity compared with the monolithic high-strength 7A62 plate (351 m/s). Energy analysis further reveals that the plastic energy dissipation of this configuration reaches 335.2 J, which is about 24% higher than that of the monolithic 7A62 plate (270.4 J).
3. The stacking sequence is a key factor influencing the ballistic performance of laminated plates. A rationally designed alternating sequence (ABAB) can significantly enhance overall performance. Energy analysis indicates that this advantage stems from the synergistic effect of multi-interface delamination on coordinated plastic energy consumption and damage dissipation.

### 5.2. Research Limitations and Model Description

This study has the following limitations, which should be addressed in future work:

1. The conclusions are derived entirely from numerical simulations. Although the material constitutive parameters and the dynamic response of the laminates under SHPB loading were validated through experimental-simulation comparisons, the performance ranking of the laminated configurations, particularly the superiority of the ABAB sequence, requires direct experimental validation through ballistic limit (V_50_) tests, which is planned as the immediate next step (see Section 5.3).
2. The research focuses on the influence of the stacking sequence (configuration effect) on ballistic performance. The thickness ratio (1:5) was predefined as a fixed parameter; its optimal value and its interaction with the stacking sequence were not explored.
3. Although the key parameters for the interfacial cohesive zone model were referenced from the literature, a parameter sensitivity analysis for these values was not conducted in this study.

### 5.3. Future Research Prospects

To deepen the mechanistic understanding and advance engineering applications, subsequent research is recommended to focus on:

1. Completing Closed-Loop Experimental Validation: Conduct ballistic limit (V_50_) tests on the baseline material (7A62) and representative optimized configurations (e.g., ABAB). Systematically compare the experimental results with simulation predictions to achieve a complete verification from material parameters to structural performance.
2. Performing Multi-Parameter Collaborative Optimization: Systematically investigate the influence of key design parameters such as the thickness ratio and interface properties (strength, toughness). Conduct multi-variable collaborative optimization in conjunction with the stacking sequence to explore globally optimal designs.
3. Extending to Complex Service Conditions: Expand the research to scenarios closer to real protective requirements, such as different projectile shapes, oblique penetration, and multiple impacts. Evaluate the universality and robustness of the configuration effects under these conditions.

### 5.4. Engineering Application Value

The stacking design criterion established in this study can be directly applied to guide the development of lightweight composite armor. This provides an effective technical pathway for significantly enhancing protective performance through structural innovation under the premise of a fixed material system, offering valuable insights for the research and development of lightweight protective components in fields such as special vehicles and aerospace.

## Figures and Tables

**Table 1:** Compositions of alloys (wt.%).

Alloys	Si	Fe	Cu	Mn	Mg	Cr	Zn	Ti	Zr	Al
7A52	0.05	0.14	0.11	0.31	2.42	0.19	4.39	0.08	0.11	Bal.
7A62	0.05	0.11	0.30	0.43	2.86	0.15	6.98	0.06	0.10	Bal.
7A01	0.05	0.14	<0.01	<0.01	<0.01	<0.01	1.02	0.01	—	Bal.

**Table 2:** Stress triaxiality and fracture strain under various stress states.

Notched Bar Specimens	η	$\varepsilon_{f}$
7A52 (R=∞)	0.33	0.71
7A52 (R=9 mm)	0.44	0.45
7A52 (R=3 mm)	0.62	0.18
7A52 (R=2 mm)	0.74	0.13
7A62 (R=∞)	0.33	0.14
7A62 (R=9 mm)	0.61	0.14
7A62 (R=3 mm)	0.74	0.12
7A62 (R=2 mm)	0.89	0.11

**Table 3:** Johnson-Cook Constitutive and Fracture Model Parameters.

Materials	*A*	*B*	*n*	*C*	*m*	$D_{1}$	$D_{2}$	$D_{3}$	$D_{4}$	$D_{5}$
7A52	368	735	0.72	0.023	0.91	0.02	1.67	2.65	0.036	0.65
7A62	583	812	0.79	0.025	0.63	0.09	0.18	6.87	0.016	12.95

**Table 4:** Material properties of the 7A01 bonding layer and parameters of the interfacial cohesive zone model.

Category	Parameter	Symbol	Value	Note/Source
Linear Elastic	Young’s Modulus	*E*	70 GPa	
	Poisson’s Ratio	*ν*	0.33	Dimensionless
Cohesive Zone Model	Normal/Shear Strength	$\tau_{n\;}^{o}{,\tau }_{s}^{o}$	94 MPa	[11]
	Critical Failure Displacement	$\delta_{c}$	0.1 mm	[11]
	Mode-I Fracture Energy	$G_{Ic}$	4.7 N/mm	$G_{Ic}=\frac{1}{2}\tau_{0}\delta_{c}$
	Mode-II Fracture Energy	$G_{IIc}$	4.7 N/mm	Assumed $G_{IIc}\;=\;G_{Ic}$
	Normal Initial Stiffness	$K_{nn}$	1 × 10^5^ N/mm^3^	Engineering value for explicit analysis stability
	Shear Initial Stiffness	$K_{ss}$	1 × 10^5^ N/mm^3^	Same as above

**Table 5:** Residual Velocities of projectile Impacting Laminates with Different Configurations.

Configurations	Initial Velocity (m/s)	Residual Velocity (m/s)
A(7A52)	828	480
B(7A62)		351
AB		363
BA		407
ABAB		256
BABA		310
BAAB		272
ABBA		305

**Table 6:** Energy dissipation for each configuration during penetration.

Configurations	Plastic Dissipation Energy (J)	Damage Dissipation Energy (J)	Sum of Plastic and Damage Dissipation Energy (J)
A(7A52)	401.0	0.9	401.9
B(7A62)	270.4	149.2	419.2
AB	324.2	133.2	457.4
BA	275.8	152.1	427.9
ABAB	335.2	144.9	480.1
BABA	310.6	145.7	456.3
BAAB	336.4	136.0	472.4
ABBA	301.6	164.1	465.7

## Data Availability

The original contributions presented in this study are included in the article. Further inquiries can be directed to the corresponding author.
